# Plasma Lutein, a Nutritional Biomarker for Development of Advanced Age-Related Macular Degeneration: The Alienor Study

**DOI:** 10.3390/nu13062047

**Published:** 2021-06-15

**Authors:** Bénédicte M. J. Merle, Audrey Cougnard-Grégoire, Jean-François Korobelnik, Wolfgang Schalch, Stéphane Etheve, Marie-Bénédicte Rougier, Catherine Féart, Cécilia Samieri, Marie-Noëlle Delyfer, Cécile Delcourt

**Affiliations:** 1 Institut National de la Santé et de la Recherche Médicale (INSERM), Université de Bordeaux, Bordeaux Population Health, F-33000 Bordeaux, France; audrey.cougnard-gregoire@u-bordeaux.fr (A.C.-G.); jean-francois.korobelnik@chu-bordeaux.fr (J.-F.K.); catherine.feart-couret@u-bordeaux.fr (C.F.); cecilia.samieri@u-bordeaux.fr (C.S.); marie-noelle.delyfer@chu-bordeaux.fr (M.-N.D.); cecile.delcourt@u-bordeaux.fr (C.D.); 2 Service d’Ophtalmologie, Centre Hospitalier Universitaire de Bordeaux, 33000 Bordeaux, France; marie-benedicte.rougier@chu-bordeaux.fr; 3 DSM Nutritional Products, 4303 Kaiserraugst, Switzerland; wolfgang.schalch@dsm.com (W.S.); stephane.etheve@dsm.com (S.E.)

**Keywords:** age-related macular degeneration, lutein, biomarker, epidemiology, nutrition, cohort, risk, population

## Abstract

Lutein and zeaxanthin may lower the risk of age-related macular degeneration (AMD). We evaluated the associations of plasma lutein and zeaxanthin with the incidence of advanced AMD in the Alienor study (Antioxydants Lipides Essentiels Nutrition et Maladies Oculaires). Alienor study is a prospective population-based cohort of 963 residents of Bordeaux, France, who were 73 years or older at baseline (2006–2008). The present study included 609 participants with complete ophthalmologic and plasma carotenoids data. Examinations were performed every two years over an eight-year period (2006 to 2017). Plasma lutein and zeaxanthin were determined at baseline from fasting blood samples using high-performance liquid chromatography. Cox proportional hazard models were used to assess associations between plasma lutein, zeaxanthin, and their (total cholesterol (TC) + triglycerides (TG)) ratios with AMD. Among the 609 included participants, 54 developed advanced incident AMD during a median follow-up time of 7.6 years (range 0.7 to 10.4). Participants with higher plasma lutein had a reduced risk for incident advanced AMD in the fully adjusted model (HR = 0.63 per 1-SD increase (95% CI, 0.41–0.97), *p* = 0.03). A similar association was observed using the lutein/(TC + TG) ratio (HR = 0.59 (95% CI, 0.39–0.90), *p* = 0.01). No associations were evidenced for other carotenoids. Higher plasma lutein was associated with a 37% reduced risk of incident advanced AMD.

## 1. Introduction

Age-related macular degeneration (AMD) is the leading cause of central vision loss in industrialized countries [1]. This degenerative disease affects the central part of the retina, which is crucial for daily living tasks such as reading, driving, and recognition of faces. Advanced forms of the disease, neovascular or atrophic AMD, associated with visual impairment, are generally preceded by early stages. While no treatment is currently available for atrophic AMD, effective but costly treatments are available for the neovascular form [2,3]. The risk of developing AMD is jointly determined by age, individual genetic background, and lifestyle (i.e., smoking, diet, sunlight exposure) [1,4]. Prevention strategies based on the modifiable risk factors of AMD, including nutrition, may help decrease the major medical and social burden associated with AMD.

Epidemiological studies have reported a reduced risk of AMD associated with high consumption of antioxidants [5,6,7,8,9], omega-3 polyunsaturated fatty acids [10], and recently, with high adherence to a healthful diet rich plant foods, fish, olive oil, and low consumption of meat and dairy products [11,12]. Among the family of carotenoids (pigments present in plant foods), lutein and zeaxanthin are highly concentrated in the macula (30 to 10,000 times higher than in others tissues), where they form the macular pigment [13]. Macular pigment has many properties within the retina; it acts as a filter by absorbing blue light, which can cause irreversible damages to the retina [14]. Macular pigment also has anti-inflammatory and antioxidant properties potentially useful against AMD, as oxidative stress and inflammation are known to be strongly involved in the pathophysiology of AMD [14,15,16]. The presence of carotenoids in the brain suggests that the macular pigment may also have an effect on neuron function [17,18,19,20]. Macular pigment could improve visual performance by attenuating chromatic aberration and light scatter.

Epidemiological studies have observed a reduced risk of AMD associated with high dietary consumption of carotenoids, including lutein and zeaxanthin [5,6,7,8,9,21,22,23,24,25,26]. However, dietary assessments represent an indirect measurement of nutritional status and are subject to many limitations. Blood biomarkers are an objective tool to assess nutritional status. A few epidemiological studies have suggested that AMD was associated with plasma carotenoids [6,7,27,28,29,30], but none of them is prospective, allowing studying the development of AMD.

We, therefore, investigated the associations between plasma lutein and zeaxanthin, a biomarker of nutritional carotenoids status, and the 8-year of incidence of advanced AMD in a population-based prospective study of French elderly subjects.

## 2. Materials and Methods

### 2.1. Study Population

The Alienor study (antioxydants, lipides essentiels, nutrition et maladies oculaires) is an ongoing prospective population-based study aiming at assessing the associations of age-related eye diseases with nutritional factors [31].

Participants were recruited from the ongoing population-based Three-City (3C) Study, which included 9294 subjects aged 65 years or more from three French cities (Bordeaux, Dijon, and Montpellier), among whom 2104 were recruited in Bordeaux [32].

The Alienor study consists of eye examinations, which are offered every two years to all participants of the 3C cohort in Bordeaux since 2006 (http://www.alienor-study.com/langue-english-1.html, accessed on 14 June 2021). In 2006–2008, 963 (66.4%) participated in the Alienor study’s baseline eye examination, out of 1450 alive 3C participants. Detailed characteristics of participants and nonparticipants have been described elsewhere [31].

Of the 963 Alienor participants, 395 were reexamined at Alienor ancillary study on macular pigment (2008–2009), 624 at the first follow-up (2009–2010), 614 at the second follow-up visit (2011–2012), 513 at the third follow-up visit (2013–2015), and 435 at the fourth follow-up visit (2015–2017). The design was approved by the Ethical Committee of Bordeaux (Comité de Protection des Personnes Sud-Ouest et Outre-Mer III, ethic approval code: 2006/10) in May 2006. All participants provided written informed consent in accordance with the Declaration of Helsinki to participate in the study.

### 2.2. Eye Examination

The eye examinations, including two 45° nonmydriatic color retinal photographs, occurred in the Department of Ophthalmology of the University Hospital of Bordeaux. [31]. More details are given in Supplementary Material S2.2.

Color fundus photographs were performed using a high-resolution digital nonmydriatic retinograph (TRC NW6S; Topcon, Japan). In addition to retinal photographs, from 2009 (first follow-up visit), a spectral-domain optical coherence tomography (SD-OCT) examination of the macula and the optic nerve was performed using Spectralis (Software Version 5.4.7.0; Heidelberg Engineering, Heidelberg, Germany).

### 2.3. AMD Classification

As previously published, retinal photographs of both eyes were graded in duplicate by two trained graders according to the international classification and to a modification of the grading scheme used in the multi-ethnic study of atherosclerosis for drusen size, location, and area [31,33,34,35]. Inconsistencies between the two graders were adjudicated by a retina specialist. All advanced AMD cases (i.e., neovascular and/or atrophic AMD) were adjudicated and confirmed by retina specialists. AMD classification is detailed in Supplementary Material S2.3.

In addition, SD-OCT macular scans (vertical and horizontal lines, macular volume) were interpreted for signs of retinal atrophy and neovascular AMD (subretinal fluid, subretinal tissue, pigment epithelium detachment, intra-retinal fluid). Finally, the classification of atrophic and neovascular AMD was based on all available information (ophthalmological history and treatments, retinal photographs, SD-OCT scans).

At each visit, each eye was classified according to one of the following exclusive groups: no AMD, early AMD1, early AMD2, advanced AMD. None of the people involved in the classification of AMD had any access to carotenoid measurements at any time of the study.

### 2.4. Incidence of AMD

Incidence of advanced AMD was defined as the eye progressing from no or early AMD at baseline eye examination (2006–2008) to advanced AMD at any time-point during the study period. The date of occurrence of advanced AMD was calculated as the midpoint of the interval between the last visit without advanced AMD and the first visit with advanced AMD. Follow-up ended at the date of occurrence of advanced AMD or the date of the last gradable examination. Participants with advanced AMD or no gradable eyes at baseline were excluded from the analysis.

For the purpose of AMD subtype analysis, neovascular AMD comprised all participants with some neovascular lesions, with or without atrophy. Atrophic AMD was defined as pure geographic atrophy (in the absence of neovascular AMD).

### 2.5. Plasma Carotenoids and Lipids Assessment

Plasma measurements were determined from fasting blood samples collected at the 3C baseline visit (1999–2001) into heparinized evacuated tubes and centrifuged at 1000 × *g* for 15 min and stored at −80 °C until determinations.

Plasma concentration of lutein zeaxanthin and other carotenoids (alpha- and beta-carotene, lycopene, beta-cryptoxanthin) were performed at DSM Nutritional Products (Kaiseraugst, Switzerland). Their concentrations were determined by normal-phase high-performance liquid chromatography (HPLC), using dedicated analytical methods [36,37]. None of the people involved in plasma carotenoids determination had any access to ocular clinical findings or genetic data at any time of the study.

Plasma lipids (high-density lipoprotein (HDL) and low-density lipoprotein (LDL)-cholesterol, triglycerides) were measured at the Biochemistry Laboratory of the University Hospital of Dijon (Dijon, France) using routine enzymatic techniques. In humans, major carriers for carotenoids are LDL and HDL [38]. There is previous evidence that the blood availability of carotenoids is dependent on the concurrent concentrations of lipids. Carotenoid/(TC + TG) ratios were computed to better consider the effect of concurrent concentrations of lipids on the bioavailability of carotenoids, as previously performed in several studies, including our cohort [38,39,40].

### 2.6. Other Variables

Genotyping was performed on DNA extracted from leukocytes at 3C study baseline (1999–2001) and kept frozen at −80 °C. Centralized facilities for genotyping were provided by the Lille Genopôle, and a genome-wide scan was performed at the Lille Genopôle [41]. The genetic risk score is based on the paper of Fritsche et al. [42]. The score corresponds to the sum of the corresponding beta multiplied by the number of minor alleles for each single nucleotide polymorphism (SNP). The betas used are calculated from the fully conditioned odds ratios in the Fritsche et al. paper [42]. Due to the high number of missing data for the three following SNPs: *TRPM3 rs71507014*, *CNN2 rs67538026*, and *MMP9 rs142450006*, these SNPs have been excluded from the risk score calculation. The present genetic risk score is based on 49 SNPs and was calculated for all participants who had available data for at least the five majors AMD-related genes (*CFH rs10922109*, *CFH rs570618*, *C2 rs11603772*, *C3 rs2230199*, and *ARMS2 rs3750846*).

Age (years), sex, marital status (married/not married), smoking (never smoker, smoker <20 pack-years (PY), smoker ≥20 PY, PY = packs (20 cigarettes) smoked per day X years of smoking) and physical activity (none, medium, high, no answer) were measured using self-reported questionnaires at 3C study baseline (1999–2001) [31]. Vascular risk factors included body mass index (BMI: weight (kg)/height (m²)) and diabetes (fasting blood glucose ≥7.0 mmol/L or non-fasting blood glucose ≥11 mmol/L or use of antidiabetic medication or self-reported diabetes). Sunlight exposure was determined by the average annual ambient ultra-violet radiation (UVR) exposure estimated using the residential history [43]. AMD nutritional supplement use (Yes/No) was evaluated from the 3C study baseline (1999–2001) to the last follow-up visit (2015–2017). Participants who reported taking vitamins, minerals, or AMD supplements at least once during this period were considered a user of AMD nutritional supplements. At the time of carotenoids measurement (3C study baseline), no participant reported taking lutein or zeaxanthin supplement.

Dietary data are detailed in Supplementary Material S2.6.

### 2.7. Statistical Analysis

Subjects excluded from analyses were compared to those included using a logistic regression model for each characteristic separately.

A few participants have very high extreme values for lutein (*n* = 4) or zeaxanthin (*n* = 5) and do not allow us to run the statistical model properly. Outliers were identified using Grubbs’s test [44]. Participants with an extremely high level of lutein were only excluded from the lutein model, and participants with an extremely high level of zeaxanthin were only excluded from the zeaxanthin model.

Each plasma carotenoid concentration and carotenoid/(total cholesterol (TC) + triglycerides (TG)) ratio were described by its mean and standard deviation (SD) among participants with incident AMD and control participants.

The associations of carotenoids and carotenoid/(TC + TG) ratios with an incidence of advanced AMD were analyzed using Cox proportional hazards models with delayed entry and age as a time scale, which allows for a better adjustment for age than the classical Cox models based on the time from entry in the study [45]. The individual eye was used as the unit of analysis [PROC PHREG with the covariance aggregate option in SAS version 9.4 (SAS Institute Inc. Cary, NC, USA), to take into account intra-individual correlation between eyes]. Model 1 was adjusted for sex and AMD grade at baseline, and model 2 was further adjusted for smoking status, alcohol consumption, the season of blood draw, BMI, diabetes, total cholesterol, triglycerides, marital status, physical activity, use of AMD supplement, and genetic risk score. Variables retained in model 2 were factors associated with the incidence of AMD and/or with carotenoids, in our study or in the literature. Hazard ratios (HRs) are for 1-SD carotenoid increase. In all Cox models, the proportional hazard assumptions and the log linearity were tested and satisfied.

#### Secondary Analyses

We also assessed whether associations of carotenoids with AMD might be independent of dietary intake and diet quality by further adjusting model 2 for dietary intake of carotene, lutein, zeaxanthin, TEI, and MeDi score. Sunlight exposure has been reported associated with AMD in epidemiological studies, including in our Alienor cohort [43]. UVR represents the most energetic part of optical radiations and, thus, is responsible for a large part of photochemical damage in the eye [46]. Since dietary habits differ according to French regions and may thus be associated with sunlight exposure, lifetime UVR exposure (not available for the whole sample) was therefore studied as a potential covariate by adding this variable to model 2.

SAS software version 9.4 (SAS Institute Inc. Cary, NC, USA) and R software version 1.3.959 (R Foundation for Statistical Computing, Vienna, Austria) were used for analyses.

## 3. Results

### 3.1. Characteristics of the SAMPLE

**Figure 1:**
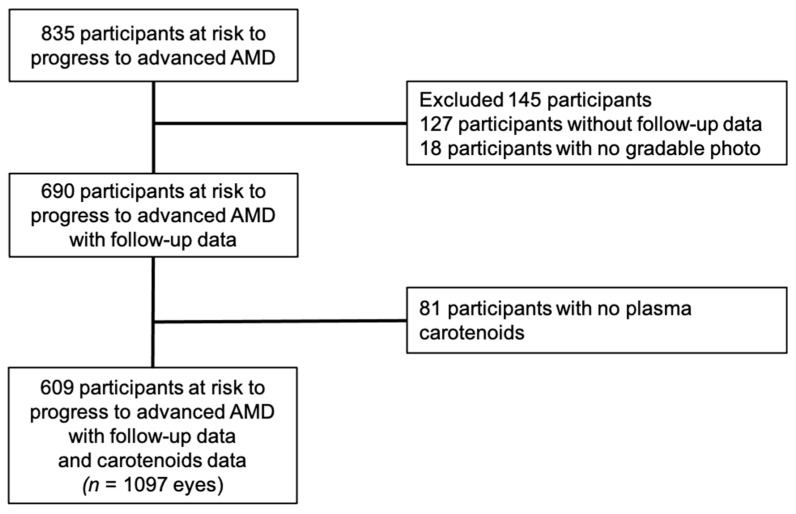
Flow chart showing selection of participants for analyses. AMD = age-related macular degeneration; Alienor = antioxydants, lipides essentiels, nutrition et maladies oculaires.

Among the 963 participants included in the Alienor study, 85 had no baseline gradable examination, 43 had advanced AMD for at least one eye, leaving 835 participants at risk to develop advanced AMD. Of the 835 participants at risk of developing advanced AMD, 127 had no follow-up examination, and 18 had no gradable examination for any follow-up visit. In addition, 81 participants were excluded because of missing data for plasma carotenoid measures (Figure 1). Thus, 609 participants free of advanced AMD at Alienor baseline eye examination with complete plasma data together with follow-up information were included in our analyses. Among the 609 included participants, 54 (8.9%) developed advanced incident AMD during a median follow-up time of 7.6 years (range, 0.7 to 10.4 years).

Participants included in the analyses tended to be more frequent users of AMD nutritional supplements and were different regarding physical activity from participants excluded. They were not different regarding other sociodemographic, lifestyle, plasma measurements, medical, genetic, or ocular data (Table 1).

### 3.2. Multivariate Associations Between Plasma Lutein and Zeaxanthin and Risk of AMD

Associations of incident advanced AMD with plasma lutein, zeaxanthin, and their ratios are shown in Table 2. After adjustment for sex and AMD grade at Alienor baseline (Model 1), higher lutein and zeaxanthin were associated with a lower risk of developing advanced AMD, with an HR of 0.71 (95% confidence interval (CI), 0.52–0.98), and 0.69 (95% CI 0.50–0.95), respectively. After further adjustments for smoking status, alcohol consumption, the season of blood draw, BMI, diabetes, TC, TG, marital status, physical activity, use of AMD supplement, and genetic risk score (Model 2), high lutein remained associated with a lower risk of AMD with an HR of 0.63 (95% CI, 0.41–0.97), but the association with zeaxanthin was no longer statistically significant (HR 0.80 (95% CI 0.55–1.17). Regarding ratios, an increase in lutein and zeaxanthin was associated with a reduced risk of advanced AMD in model 1. In the fully adjusted model 2, only lutein/(TC + TG) ratio remained associated with AMD (HR = 0.59 (95% CI, 0.39–0.90).

### 3.3. Multivariate Associations Between Plasma Lutein and Zeaxanthin and Risk of Neovascular and Atrophic AMD

Table 3 shows the associations between plasma lutein, zeaxanthin and their ratios and incidence of advanced neovascular and atrophic AMD separately. HRs for neovascular and atrophic AMD separately were similar but not statistically significant to those reported for any AMD.

### 3.4. Multivariate Associations Between Other Carotenoids and Risk of AMD

Table 4 displays the associations between other carotenoids and their ratios and incidence of advanced AMD. No other carotenoids nor ratios were associated with AMD.

### 3.5. Secondary Analyses

In secondary analyses, to assess whether these associations were independent of diet, we further adjusted for dietary intake of carotenes, lutein and zeaxanthin, TEI, and MeDi score (Table 5). Associations between lutein and lutein/(TC + TG) ratio with AMD were similar for advanced AMD, as well as for neovascular and atrophic, separately. In a subsample (*n* = 786 eyes), we further adjusted for sunlight exposure, and the results remained unchanged. No interaction between carotenoids and sunlight exposure was found (data not shown).

## 4. Discussion

In the present longitudinal study, higher plasma lutein at baseline was associated with a 37% reduced risk of incident advanced AMD over a period of 7 years. A similar association was observed with the lutein/(TC + TG) ratio, with a 41% reduced risk of incident advanced AMD in participants with higher ratios. None of the other carotenoids nor carotenoid/(TC + TG) ratios were associated with the incidence of advanced AMD, highlighting the importance of lutein for the retina. Similar trends were observed with atrophic and neovascular AMD forms separately.

By evaluating the associations of plasma carotenoid concentrations with an incidence of advanced AMD, this prospective study expands on prior cross-sectional and case-control studies on this topic. Visual impairment due to AMD may influence nutritional practices linked to plasma status; prospective studies, by assessing nutritional status prior to the onset of the disease, limits such reverse causation. Thus, the prospective design is more accurate and less biased than a cross-sectional or case-control design to evaluate the association between nutritional status and AMD risk. Moreover, dietary assessment methods rely on the participants’ memory and perceptions and face the difficulties of the high variability and complexity of the human diet, the bias in reporting due to social standards and nutritional recommendations, and the estimation of the nutritional content of foods. A strength of this study is the use of nutritional blood biomarkers, which represent a more precise and objective alternative for the assessment of nutritional status. Plasma lutein has been evaluated in numerous studies, showing suitable correlations with diet and sensitivity in a change in supplementation studies [47,48,49,50]. In our study plasma lutein+zeaxanthin was correlated to dietary intake of lutein+zeaxanthin (*r* = 0.21 *p* < 0.0001) and to Mediterranean diet score (*r* = 0.24 *p* < 0.0001). Lutein supplementation has also been reported to increase MPOD [51,52,53].

Our results are consistent with previous cross-sectional and case-control studies that have reported higher plasma lutein+zeaxanthin in participants with advanced AMD [27,28,30] or any AMD [6,7,29]. All these studies showed results in the same direction, but only an American case-control [27] and the French study POLA [7] showed protective associations for lutein+zeaxanthin. Two studies [7,29] showed protective associations for zeaxanthin; associations for lutein were in the same direction but did not reach statistical significance. In the present study, only high lutein was significantly associated with decreased incidence of AMD; this could be explained by the lower variability in zeaxanthin in our sample or/and the relatively low number of incident cases.

Our findings also confirm prospective studies based on dietary intake reporting that participants having a higher dietary intake of lutein+zeaxanthin had a reduced risk for developing advanced AMD [8,21,24,25,54].

In the retina, the two main mechanisms by which lutein might protect against AMD are its ability to absorb blue light and to counteract free radicals [14]. By absorbing light in the blue range, which is particularly damaging to the retina, lutein may protect against light damages. Another function of lutein that might explain its possible protective functions is its ability to counteract free radicals or reactive oxygen species, thereby acting as an antioxidant. Lutein possessing a series of unconjugated double bonds that are believed to be very effective antioxidants [55]. Reactive oxygen species are particularly high in the retina, and oxidative stress is involved in AMD pathogenesis. Another important role of lutein and zeaxanthin is their neural efficiency suggested by their presence in the brain [17,18,19,20]. Plasma carotenoids have been associated with reduced risk for dementia [39], and studies have suggested a protective effect in neuropathological processes [56] and a potential anti-amyloid effect of beta-carotene [57], extending the interest of carotenoids beyond ocular health.

Regarding subtypes, HRs for lutein, zeaxanthin and their ratios were similar for both forms of AMD, but these associations were not statistically significant, which might be explained by a relatively low statistical power due to the small number of incident cases for each subtype.

The originality of the present study is to study plasma lutein and zeaxanthin as a function of plasma lipids, which we previously did for studying carotenoids concentrations in relation to dementia [39]. The transport and absorption of carotenoids, lipophilic components, are closely linked to cholesterol metabolism. Plasma carotenoids concentrations are strongly correlated with the concentration of plasma cholesterol et triglyceride, which cannot be neglected when assessing nutritional status in carotenoids [14,58,59]. Thus, the carotenoid/(TC + TG) ratio should be considered as a more accurate indicator of actual carotenoid plasma status than carotenoid concentrations per se. By showing a stronger association for ratio than lutein alone, our results are in line with this statement.

In observational studies, residual confounding is always a concern. In the present study, results were similar in the basic model (unadjusted model) and the fully adjusted model, suggesting that our results are not highly confounded. In secondary analyses, we further adjusted for dietary intakes of carotenes, lutein and zeaxanthin, and adherence to the MeDi score. Those results did not differ from main analyses suggesting that plasma level and dietary intake are independently associated with AMD in our study.

In addition, our findings are based on prospective follow-up, thereby limiting reverse causation. However, only randomized clinical trials can prove the causal nature of the associations. The Age-Related Eye Diseases Study (AREDS) clinical trial suggested that the addition of lutein+zeaxanthin, DHA+EPA, or both to the AREDS formulation in primary analyses did not further reduce the risk of progression to advanced AMD [60]. However, because of the potential increased incidence of lung cancer in former smokers, lutein+zeaxanthin could be more appropriate than beta-carotene in the AREDS-type supplement [61]. Moreover, among participants who had the lowest dietary intake of lutein and zeaxanthin, those who took AREDS with lutein+zeaxanthin had a 26% lower risk of progressing to advanced AMD compared to participants taking the original AREDS formula.

Selection bias cannot be completely dismissed in our study, as participants included in this analysis were different from nonparticipants. However, this selection bias is limited, as they differed only regarding physical activity and AMD supplementation.

Other limitations of our study include a single measurement of plasma carotenoids that does not allow us to measure changes over time. However, previous analyses have reported high reproducibility across repeated measurements [62]. This measurement error is likely to be independent of AMD status and most probably induces a bias toward null in the estimation of the association of AMD with plasma carotenoids. If we had several measurements of carotenoids, the association of plasma lutein with incident AMD would thus probably be stronger. Nevertheless, we observed that a single carotenoid measurement performed 7 years before the evaluation of AMD was associated with the risk of AMD, suggesting that exposure could influence AMD progression in the long term. The relatively small number of incident cases might be one of the reasons for the absence of statistical significance of some of the associations (in particular with zeaxanthin).

## 5. Conclusions

In conclusion, this prospective study evidenced an association of high plasma lutein, a reliable biomarker of the nutritional status of lutein, with a lower incidence of advanced AMD. In the future, such nutritional biomarkers could be useful for the identification of individuals with a deficient nutritional status for AMD and monitoring nutritional interventions in combination with other nutritional biomarkers and an evaluation of the diet quality.

## Figures and Tables

**Table 1:** Baseline sociodemographic, lifestyle, medical, ocular and genetic characteristics according to participants included and excluded from analyses. Alienor study, 2006–2017.

Characteristics	Included (*n* = 609)	Excluded (*n* = 226)	*p*-Value
	Mean ± SD or *n* (%)	Mean ± SD or *n* (%)	
Age (years)	79.7 ± 4.4	80.3 ± 4.2	0.07
Sex	*N* = 609	*N* = 226	0.23
Men	223 (36.6)	93 (41.2)	
Women	386 (63.4)	133 (58.8)	
Marital status	*N* = 609	*N* = 226	0.40
Married	369 (60.6)	144 (63.7)	
Not married	240 (39.4)	82 (36.3)	
Smoking (pack-year)	*N* = 605	*N* = 222	0.97
Never smoker	395 (65.3)	143 (64.4)	
<20	106 (17.5)	40 (18.0)	
≥20	104 (17.2)	39 (17.6)	
Physical activity	*N* = 609	*N* = 226	<0.0001
None	336 (55.1)	102 (45.1)	
Medium	124 (20.4)	42 (18.6)	
High	65 (10.7)	19 (8.4)	
No answered	84 (13.8)	63 (27.9)	
Dietary intake	*N* = 593	*N* = 201	
Alcohol (g/d)	12.7 ± 16.9	14.2 ± 6.3	0.29
Carotenes (µg/d)	3894 ± 5705	3383 ± 4650	0.44
Lutein and zeaxanthin (µg/d)	1022 ± 2852	658 ± 1263	0.20
Total energy intake (kcal/d)	1709 ± 546	1727 ± 500	0.91
Mediterranean diet score	*N* = 573	*N* = 197	0.35
Low (0–3)	183 (31.9)	74 (37.6)	
Medium (4–5)	251 (43.8)	79 (40.1)	
High (6–9)	139 (24.3)	44 (22.3)	
AMD nutritional supplement during the study period	*N* = 609	*N* = 226	0.002
No	536 (88.0)	216 (95.6)	
Yes	73 (12.0)	10 (4.4)	
Body mass index (kg/m²)	*N* = 608	*N* = 222	0.67
<25	237 (39.0)	79 (35.6)	
(25–30)	282 (46.4)	108 (48.6)	
≥30	89 (14.6)	35 (15.8)	
Diabetes	*N* = 604	*N* = 172	0.34
No	558 (92.4)	155 (90.1)	
Yes	46 (7.6)	17 (9.9)	
Plasma lipids (mmol/L)	*N* = 609	*N* = 175	
Total cholesterol	5.81 ± 0.96	5.73 ± 0.98	0.48
LDL	3.67 ± 0.84	3.57 ± 0.84	0.43
HDL	1.59 ± 0.39	1.59 ± 0.37	0.94
Triglycerides	1.22 ± 0.58	1.23 ± 0.60	0.93
Genetic risk score	*N* = 530	*N* = 157	0.82
	0.25 ± 1.23	0.28 ± 1.04	
AMD grade at baseline	*N* = 609	*N* = 226	0.36
No AMD	415 (68.1)	165 (73.0)	
Early AMD1	121 (19.9)	40 (17.7)	
Early AMD2	73 (12.0)	21 (9.3)	
Plasma carotenoids (mmol/L)	*N* = 609	*N* = 138	
Lutein	0.30 ± 0.15	0.28 ± 0.15	0.15
Zeaxanthin	0.07 ± 0.04	0.07 ± 0.04	0.66
Beta-cryptoxanthin	0.31 ± 0.24	0.28 ± 0.22	0.22
Alpha-carotene	0.19 ± 0.15	0.19 ± 0.22	0.81
Beta-carotene	0.76 ± 0.55	0.75 ± 0.72	0.83
Lycopene	0.47 ± 0.32	0.46 ± 0.31	0.83
Total carotenoids	2.09 ± 1.02	2.05 ± 1.28	0.69

AMD: age-related macular degeneration; SD: standard deviation.

**Table 2:** Associations between plasma lutein, zeaxanthin, and their (TC + TG) ratios and incidence of advanced age-related macular degeneration. Alienor study, 2006–2017.

Plasma Carotenoids	Non-Incident AMD		Incident AMD		Model 1 ^a^			Model 2 ^b^		
	*n*	Mean ± SD	*n*	Mean ± SD	*n* Cases/Total (Eye)	HR (95% CI)	*p*-Values	*n* Cases/Total (Eye)	HR (95% CI)	*p*-Values
Lutein, (mmol/L)	552	0.30 ± 0.15	53	0.29 ± 0.14	79/1089	0.71 (0.52, 0.98)	0.037	66/869	0.63 (0.41, 0.97)	0.03
Zeaxanthin, (mmol/L)	551	0.07 ± 0.04	53	0.07 ± 0.04	78/1087	0.69 (0.50, 0.95)	0.02	65/867	0.82 (0.56, 1.21)	0.33
Lutein/(TC + TG) ^c^	552	0.04 ± 0.02	53	0.04 ± 0.02	79/1089	0.65 (0.47, 0.89)	0.007	66/869	0.59 (0.39, 0.90)	0.01
Zeaxanthin/(TC + TG) ^c^	551	0.01 ± 0.006	53	0.01 ± 0.006	78/1087	0.69 (0.51, 0.94)	0.02	65/867	0.80 (0.55, 1.17)	0.25

^a^ Model 1, HR was estimated using Cox proportional model adjusted for sex, AMD grade at baseline. HR for 1-SD increase. ^b^ Model 2, HR was estimated using Cox proportional model adjusted for sex, AMD grade at baseline, smoking status, alcohol consumption, season of blood draw, body mass index, diabetes, total cholesterol, triglycerides, marital status, physical activity, use of AMD supplement and genetic risk score. HR for 1-SD increase. ^c^ These models were no longer adjusted for TC and TG concentrations. AMD: age-related macular degeneration; CI: confidence intervals; HR: hazards ratios; TC: total cholesterol; TG: triglycerides; SD: standard deviation.

**Table 3:** Associations between plasma lutein, zeaxanthin, and their (TC + TG) ratios and incidence of neovascular and atrophic age-related macular degeneration. Alienor study, 2006–2017.

Plasma Carotenoids	Neovascular AMD			Atrophic AMD		
	*n* Cases/Total (Eye)	HR ^a^ (95% CI)	*p*-Values	*n* Cases/Total (Eye)	HR ^a^ (95% CI)	*p*-Values
Lutein, (mmol/L)	34/869	0.64 (0.30, 1.34)	0.23	38/869	0.64 (0.41, 1.01)	0.05
Zeaxanthin, (mmol/L)	33/867	0.91 (0.54, 1.54)	0.73	38/867	0.78 (0.45, 1.38)	0.40
Lutein/(TC + TG) ^b^	34/869	0.55 (0.25, 1.18)	0.12	38/869	0.65 (0.41, 1.05)	0.08
Zeaxanthin/(TC + TG) ^b^	33/867	0.81 (0.49, 1.33)	0.40	38/867	0.85 (0.51, 1.44)	0.56

^a^ HR was estimated using Cox proportional model adjusted for sex, AMD grade at baseline, smoking status, alcohol consumption, season of blood draw, body mass index, diabetes, total cholesterol, triglycerides, marital status, physical activity, use of AMD supplement, and genetic risk score. HR for 1-SD increase. ^b^ These models were no longer adjusted for TC and TG concentrations. AMD: age-related macular degeneration; CI: confidence intervals; HR: hazards ratios; TC: total cholesterol; TG: triglycerides; SD: standard deviation.

**Table 4:** Associations between other plasma carotenoids and carotenoids/(TC + TG) ratios and incidence of advanced age-related macular degeneration. Alienor study, 2006–2017.

Plasma Carotenoids (mmol/L)	Non-Incident AMD		Incident AMD		Model 1 ^a^			Model 2 ^b^		
	*n*	Mean ± SD	*n*	Mean ± SD	*n* Cases/Total (Eye)	HR (95% CI)	*p*-Values	*n* Cases/Total (Eye)	HR (95% CI)	*p*-Values
Alpha-carotene	543	0.18 ± 0.13	52	0.17 ± 0.14	80/1097	0.82 (0.53, 1.26)	0.36	67/877	0.85 (0.55, 1.29)	0.44
Beta-carotene	543	0.75 ± 0.51	54	0.70 ± 0.38	80/1097	0.83 (0.63, 1.09)	0.18	67/877	0.92 (0.64, 1.31)	0.63
Lycopene	548	0.46 ± 0.29	54	0.41 ± 0.23	80/1097	0.79 (0.60, 1.05)	0.11	67/877	0.74 (0.51, 1.09)	0.12
Beta-cryptoxanthin	539	0.30 ± 0.20	53	0.29 ± 0.19	80/1097	0.99 (0.81, 1.23)	0.99	67/877	1.32 (1.00,1.72)	0.05
Total carotenoids	542	2.08 ± 0.96	54	1.96 ± 0.81	80/1097	0.78 (0.58, 1.03)	0.08	67/877	0.84 (0.55, 1.28)	0.41
Plasma carotenoids/(TC + TG) ratios ^c^										
Alpha-carotene	543	0.03 ± 0.02	52	0.02 ± 0.02	80/1097	0.83 (0.53, 1.32)	0.44	67/877	0.89 (0.60, 1.31)	0.55
Beta-carotene	543	0.11 ± 0.08	54	0.10 ± 0.06	80/1097	0.84 (0.63, 1.11)	0.21	67/877	0.94 (0.67, 1.33)	0.74
Lycopene	548	0.07 ± 0.04	54	0.06 ± 0.03	80/1097	0.79 (0.59, 1,02)	0.07	67/877	0.78 (0.54, 1.12)	0.18
Beta-cryptoxanthin	539	0.04 ± 0.03	53	0.04 ± 0.03	80/1097	1.04 (0.82, 1.32)	0.76	67/877	1.32 (1.00, 1.73)	0.05
Total carotenoids	542	0.30 ± 0.14	54	0.28 ± 0.12	80/1097	0.78 (0.58, 1.04)	0.09	67/877	0.88 (0.59, 1.31)	0.52
Plasma lipids (mmol/L) ^c^										
Total cholesterol	555	5.80 ± 0.94	54	5.89 ± 1.11	80/1097	1.00 (0.78, 1.29)	0.99	67/877	0.99 (0.71, 1.38)	0.96
Triglycerides	555	1.22 ± 0.58	54	1.24 ± 0.53	80/1097	1.25 (1.00, 1.56)	0.05	67/877	1.25 (0.89, 1.77)	0.21

^a^ Model 1, HR was estimated using Cox proportional model adjusted for sex, AMD grade at baseline. HR for 1-SD increase. ^b^ Model 2, HR was estimated using Cox proportional model adjusted for sex, AMD grade at baseline, smoking status, alcohol consumption, season of blood draw, body mass index, diabetes, total cholesterol, triglycerides, marital status, physical activity, use of AMD supplement and genetic risk score. HR for 1-SD increase. ^c^ These models were no longer adjusted for TC and TG concentrations. AMD: age-related macular degeneration; CI: confidence intervals; HR: hazards ratios; TC: total cholesterol; TG: triglycerides; SD: standard deviation.

**Table 5:** Associations between plasma carotenoids and carotenoids/(TC + TG) ratios and incidence of advanced AMD: sensitivity analyses adjusted for dietary intakes. Alienor scheme 2006.

Plasma Carotenoids	Advanced AMD		Neovascular AMD		Atrophic AMD	
	HR (95% CI) ^a^	*p*-Value	HR (95% CI) ^a^	*p*-Value	HR (95% CI) ^a^	*p*-Value
Lutein	0.54 (0.36, 0.82)	0.003	0.50 (0.29, 0.88)	0.02	0.62 (0.39, 1.00)	0.05
Zeaxanthin	0.77 (0.53, 1.14)	0.19	0.87 (0.47, 1.61)	0.66	0.71 (0.39, 1.27)	0.24
Alpha-carotene	0.82 (0.53, 1.27)	0.38	1.26 (0.87, 1.83)	0.22	0.37 (0.07, 1.96)	0.24
Beta-carotene	0.86 (0.59, 1.24)	0.42	1.23 (0.74, 2.03)	0.42	0.55 (0.21, 1.41)	0.21
Lycopene	0.72 (0.49, 1.07)	0.10	0.69 (0.41, 1.17)	0.17	0.77 (0.42, 1.41)	0.40
Beta-cryptoxanthin	1.25 (0.94, 1.68)	0.13	1.84 (1.27, 2.69)	0.002	0.90 (0.48, 1.67)	0.73
Total carotenoids	0.76 (0.49, 1.16)	0.20	1.24 (0.67, 2.31)	0.50	0.48 (0.20, 1.16)	0.10
Plasma carotenoids/(TC + TG) ratio ^b^						
Lutein	0.51 (0.33, 0.77)	0.002	0.44 (0.24, 0.80)	0.007	0.63 (0.39, 1.03)	0.07
Zeaxanthin	0.75 (0.52, 1.09)	0.13	0.77 (0.43, 1.39)	0.39	0.78 (0.47, 1.31)	0.35
Alpha-carotene	0.87 (0.58, 1.30)	0.49	1.24 (0.88, 1.74)	0.23	0.45 (0.08, 2.66)	0.38
Beta-carotene	0.89 (0.62, 1.27)	0.52	1.26 (0.80, 1.99)	0.32	0.60 (0.26, 1.41)	0.25
Lycopene	0.77 (0.53, 1.11)	0.16	0.77 (0.45, 1.29)	0.32	0.81 (0.47, 1.38)	0.44
Beta-cryptoxanthin	1.27 (0.94, 1.70)	0.11	1.69 (1.14, 2.49)	0.009	1.05 (0.58, 1.91)	0.86
Total carotenoids	0.80 (0.53, 1.20)	0.28	1.23 (0.70, 2.14)	0.47	0.57 (0.25, 1.28)	0.17
Plasma lipids ^b^						
Total cholesterol	0.96 (0.69, 1.33)	0.79	1.33 (0.71, 2.47)	0.37	0.81 (0.56, 1.19)	0.29
Triglycerides	1.26 (0.88, 1.80)	0.21	1.39 (0.87, 2.23)	0.17	1.09 (0.55, 2.16)	0.81

^a^ HR was estimated using Cox proportional model adjusted for sex, AMD grade at baseline, smoking status, alcohol consumption, season of blood draw, body mass index, diabetes, total cholesterol, triglycerides, marital status, physical activity, use of AMD supplement, genetic risk score, dietary intake of carotene, lutein and zeaxanthin and Mediterranean diet score. HR for 1-SD increase. ^b^ These models were no longer adjusted for TC and TG concentrations. AMD: age-related macular degeneration; CI: confidence intervals; HR: hazards ratios; TC: total cholesterol; TG: triglycerides; SD: standard deviation.

## Data Availability

The dataset presented in this article are not readily available because of ethical and legal restrictions. Requests to access the dataset should be directed to the Steering Committee of the Alienor study (contact cecile.delcourt@u-bordeaux.fr, accessed date 14 June 2021).

## Supplementary Materials

The following are available online at https://www.mdpi.com/article/10.3390/nu13062047/s1, Material S2.2 Eye Examination, Material S2.3 AMD Classification, Material S2.6 Other Variables.
