# Supplementary material for: Plasma Lutein, a Nutritional Biomarker for Development of Advanced Age-Related Macular Degeneration: The Alienor Study
Source: Nutrients. 2021 Jun 15;13(6):2047. doi: 10.3390/nu13062047 (PMC8232705; doi:10.3390/nu13062047)
Supplement: Supplementary file 1 [file nutrients-13-02047-s001.zip › nutrients-1235850-supplementary.pdf]

Supplementary Materials

S2.2 Eye Examination

The eye examinations, took place in the Department of Ophthalmology of the University Hospital of Bordeaux. They included a recording of ophthalmic history, measures of visual acuity, refraction, two 45° nonmydriatic color retinal photographs (one centered on the macula, the other centered on the optic disc), measures of intraocular pressure and central corneal thickness, and a tear breakup time test [1].

In addition, at the second and following visits, for participants who were not able to come to the hospital, the eye examination took place at home and 40° retinal photographs were taken using a digital non-mydriatic portable retinograph (Optomed Smartscope M5). All SD-OCT image capture and image assessments were performed by the same experienced technician.

S2.3 AMD Classification

Neovascular AMD included serous or hemorrhagic detachment of the retinal pigment epithelium (RPE) or sensory retina, subretinal or sub-RPE hemorrhages, and fibrous scar tissue. Geographic atrophy was defined as a discrete area of retinal depigmentation, 175 µm in diameter or larger, characterized by a sharp border and the presence of visible choroidal vessels.

Early AMD 1 was characterized by the presence of soft distinct drusen without pigmentary abnormalities or pigmentary abnormalities without large drusen (>125 µm). Early AMD 2 was characterized by the presence of soft indistinct drusen and/or reticular pseudodrusen and/or soft distinct drusen associated with pigmentary abnormalities (hyper- or hypopigmentation).

Soft distinct and indistinct drusen were larger than 125 µm in diameter and with uniform density and sharp edges or decreasing density from the center outwards and fuzzy edges, respectively. Pigmentary abnormalities were defined as areas of hyperpigmentation and/or hypopigmentation (without visibility of choroidal vessels). No AMD was defined by the absence of early AMD and advanced AMD.

In addition, SD-OCT macular scans (vertical and horizontal lines, macular volume) were interpreted for signs of retinal atrophy and neovascular AMD (subretinal fluid, subretinal tissue, pigment epithelium detachment, intra-retinal fluid). Finally, classification of atrophic and neovascular AMD were based on all available information (ophthalmological history and treatments, retinal photographs, SD-OCT scans).

S2.6 Other Variables

Dietary data. Participants were visited at home by a specifically trained dietician who administered a 148-items validated food frequency questionnaire (FFQ) and a 24-hour dietary recall [2]. The 24-hour recall was used to estimate intake in carotene, lutein, zeaxanthin and total energy intake (TEI, kcal/day) and to compute the monounsaturated

fatty acids (MUFAs) to saturated fatty acids (SFAs) ratio. Adherence to the Mediterranean diet (MeDi) was assessed using the FFQ and the MeDi score developed by Trichopoulou et al.[3] including nine components: vegetables, fruits, legumes, cereals, fish, meat, dairy products, alcohol and the MUFAs-to-SFAs ratio. Subjects were classified according to three categories: low (0–3), medium (4–5), or high adherence (6–9).

References

1. Delcourt, C.; Korobelnik, J.F.; Barberger-Gateau, P.; Delyfer, M.N.; Rougier, M.B.; Le Goff, M.; Malet, F.; Colin, J.; Dartigues, J.F. Nutrition and age-related eye diseases: the Alienor (Antioxydants, Lipides Essentiels, Nutrition et maladies OculaiRes) Study. *J. Nutr. Health Aging* **2010**, *14*, 854-861, doi:10.1007/s12603-010-0131-9.
2. Feart, C.; Jutand, M.A.; Larrieu, S.; Letenneur, L.; Delcourt, C.; Combe, N.; Barberger-Gateau, P. Energy, macronutrient and fatty acid intake of French elderly community dwellers and association with socio-demographic characteristics: data from the Bordeaux sample of the Three-City Study. *Br. J. Nutr.* **2007**, *98*, 1046-1057, doi:10.1017/S0007114507756520.
3. Trichopoulou, A.; Costacou, T.; Bamia, C.; Trichopoulos, D. Adherence to a Mediterranean diet and survival in a Greek population. *N. Engl. J. Med.* **2003**, *348*, 2599-2608, doi:10.1056/NEJMoa025039.
